# The bibliometric analysis of research on traditional Chinese medicine regulating gut microbiota for cancer treatment from 2014 to 2024

**DOI:** 10.1186/s41065-025-00456-x

**Published:** 2025-06-03

**Authors:** Youfeng Lei, Yueqin Shan, Danfeng Zhou, Chunyan Chen

**Affiliations:** 1 Department of Pharmacy, Shanghai Public Health Clinical Center, Fudan University, Shanghai, China; 2 Department of Pharmacy, Jinshan Hospital of Fudan University, Shanghai, China

**Keywords:** Bibliometric, Traditional Chinese medicine, Gut microbiota, Cancer, Cancer treatment

## Abstract

**Objective:**

The modulation of gut microbiota by Traditional Chinese Medicine (TCM) offers a promising approach to cancer treatment. However, a comprehensive bibliometric evaluation of this emerging field is lacking.

**Objective:**

This study aimed to systematically analyze global research trends, hotspots, and future directions related to TCM regulation of gut microbiota in cancer therapy from 2014 to 2024.

**Methods:**

Publications were retrieved from the Web of Science Core Collection. Bibliometric and visual analyses were conducted using VOSviewer and CiteSpace to examine publication trends, country and institutional collaborations, core authors and journals, keyword co-occurrence, and research frontiers.

**Results:**

A total of 340 relevant articles were identified. The number of publications increased significantly after 2018, indicating growing interest in this field. China dominated the research landscape, both in productivity and institutional collaboration. Core research hotspots included “short-chain fatty acids,” “tumor microenvironment,” “apoptosis,” and “immune response.” Thematic evolution analysis highlighted a shift from general gut microbiota research to precise molecular mechanisms and targeted regulation. Emerging topics such as “metabolomics” and “immune checkpoint blockade” suggest future directions.

**Conclusion:**

This study provides a comprehensive overview of the current research landscape on TCM-modulated gut microbiota in cancer treatment. By identifying core contributors, research hotspots, and frontiers, it offers valuable guidance for future investigations and interdisciplinary collaborations in this promising field.

**Supplementary Information:**

The online version contains supplementary material available at 10.1186/s41065-025-00456-x.

## Introduction

Cancer imposes a tremendous health and socioeconomic burden worldwide, posing a particularly severe challenge for many countries, including China and the United States [1, 2]. Most cancers not only place significant economic strain on families but can also be fatal [3, 4], making cancer one of the leading causes of death globally [5]. It is estimated that in 2022 there were approximately 20 million new cancer cases and 9.7 million cancer-related deaths worldwide; by 2050, the number of new cases is projected to rise to 35 million—a 77% increase compared to 2022 [6, 7]. Among these, low Human Development Index (HDI) countries are expected to experience an increase of up to 142% in new cancer cases, while medium-HDI countries are projected to see a 64% rise [7]. This trend indicates that the global cancer burden will significantly intensify in the coming decades, particularly in low- and middle-income nations. Consequently, enhancing cancer prevention, early screening, and treatment measures in these regions is of critical public health importance [8].

Currently, the primary treatment strategies for cancer include surgery, chemotherapy, radiotherapy, targeted therapy, and immunotherapy [9–11]. However, these conventional approaches are associated with several limitations [12]. For instance, chemotherapy typically requires high doses of drugs that have limited bioavailability and a narrow therapeutic index, often leading to multidrug resistance, off-target effects, and damage to normal cells [13, 14]. Although radiotherapy plays a vital role in cancer treatment, its side effects—such as radiation necrosis, moyamoya syndrome, sensory neurotoxicity, and cerebral edema—cannot be overlooked [15]. Consequently, the development of novel cancer treatment strategies has become an urgent medical need.

With continuous advancements in biomedical technology, the role of the gut microbiota in maintaining human health and in disease pathogenesis has gradually garnered attention [16, 17]. The gut microbiota, comprising symbiotic bacteria, opportunistic pathogens, and pathogenic bacteria, contains over three million genes and produces a vast array of metabolites [18]. Research indicates that imbalances in the gut microbiota are closely associated with the development of colorectal cancer and may influence the progression of other cancers—such as liver, lung, and breast cancer—via the “gut–liver” and “lung–gut” axes [16, 19].

Against this backdrop, traditional Chinese medicine (TCM) offers a novel approach for cancer research. As an important legacy of Chinese civilization, TCM has developed a unique theoretical framework over thousands of years of practice and evolution [20]. In recent years, the advantages of TCM in cancer treatment have become increasingly evident, particularly in reducing adverse reactions, providing personalized treatment, and enhancing immune function [21, 22]. Compared with conventional chemoradiotherapy, herbal medicine typically employs natural compounds with low toxicity and minimal side effects, offering high tolerability and improved quality of life for patients [23–25].

Most TCM treatments are administered orally, and as such, these medicines can significantly alter the gut microbiota upon ingestion, thereby modifying the intestinal microenvironment to effectively combat cancer [26]. For instance, recent studies have demonstrated that the traditional Chinese medicine Piwei Huang can inhibit the proliferation of colorectal cancer (CRC) cells through the suppression of the Wnt/β-catenin signaling pathway in vitro [27]. Ginsenosides, under the influence of gut microbiota, can be converted into glycogen, which plays an active role in improving the therapeutic outcomes of lung cancer [28]. In addition, mounting evidence has demonstrated that the gut microbiota plays a pivotal role in cancer development, progression, and response to treatment [29]. TCM is low in toxicity and acts on multiple targets [30]. It may fight cancer by regulating gut microbiota, especially in digestive system cancers [31].

Despite this growing body of research, no bibliometric study to date has systematically mapped the evolution, hotspots, and future directions of TCM-based modulation of gut microbiota for cancer treatment. Our study aims to fill this gap by offering a comprehensive bibliometric and visual analysis of global research output from 2014 to 2024. In addition to summarizing key trends in the field, we also highlight translational potential, as understanding the interplay between TCM and the gut microbiome could lead to more individualized, accessible, and integrative therapeutic strategies in oncology. This analysis not only informs future academic research but also has important implications for public health policy and clinical innovation.

## Materials and methods

### Data collection

The data used in this study were retrieved and downloaded from the Web of Science Core Collection (WoSCC) (Guangxi Medical University purchase edition) on January 15, 2025. The search formula used is shown in Annex 1.

**Fig. 1:**
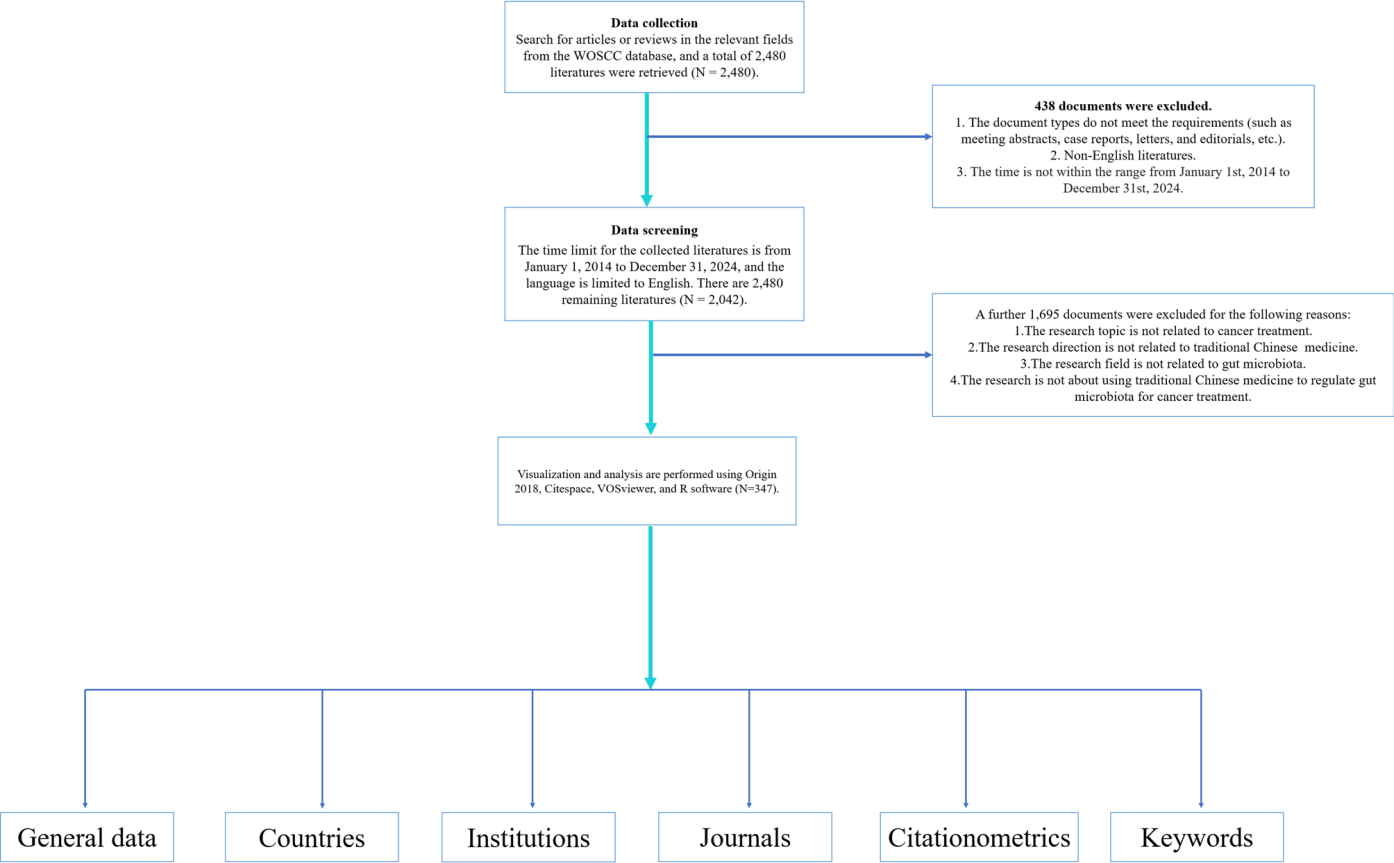
The overview of the data processing procedure

After excluding irrelevant publications, a total of 347 articles were identified (with duplicates removed). The retrieved articles were saved in plain text format and exported along with their cited references as complete records. A detailed overview of the data processing procedure is depicted in Fig. 1.

### Data analysis

To analyze annual publication trends, this study employed Origin 2018 for data visualization. Additionally, the bibliometrix package (version 4.0, available at http://www.bibliometrix.org) within R software (version 3.6.3) was used to analyze the bibliometric data [32]. Meanwhile, VOSviewer (version 1.6.17) [33] and CiteSpace (version 6.1.4) [34] were utilized to construct scientific knowledge maps, enabling us to explore research hotspots and their evolution.

To ensure the accuracy and reliability of the data, two independent authors conducted the data extraction and analysis management. VOSviewer was primarily used for the following analyses: (a) Co-authorship network analysis of countries/institutions: A threshold was set requiring a minimum of 2 publications per country or institution.(b) Co-citation analysis of sources: A minimum of 25 citations was required.(c) Keyword co-occurrence analysis: Keywords had to appear at least 3 times. Additionally, high-frequency yet generic keywords such as traditional Chinese medicine, cancer, gut microbiota, and tumor were excluded to enhance the specificity of the analysis. Furthermore, the impact factors (IFs) of the journals involved in this study were sourced from the 2024 edition of the Journal Citation Reports (JCR).

## Results

### Overview of selected studies

**Fig. 2:**
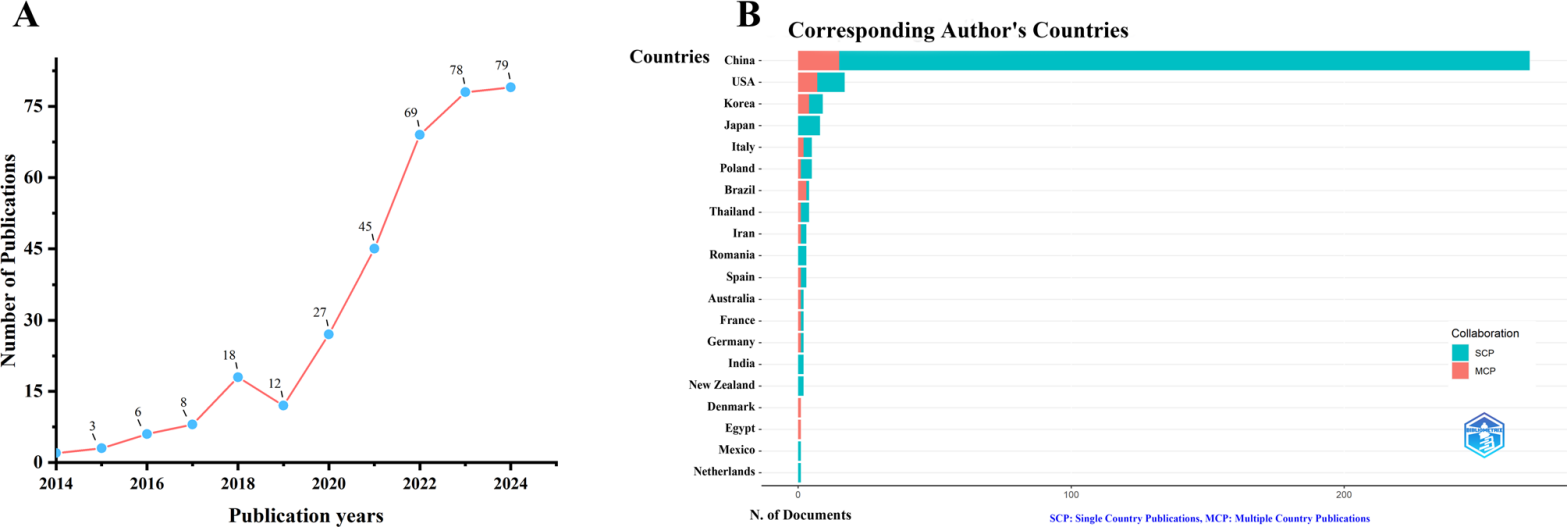
Trends in Annual Publication Outputs on the Role of TCM in Cancer Treatment via Gut Microbiota Modulation from 2014 to 2024. (**A**) Trends of annual publication outputs. (**B**) Distribution of corresponding authors’ countries and cooperation

This study retrieved and collected 347 unique publications from the WoSCC database. As shown in Fig. 2A, research on the treatment of cancer by modulating the gut microbiota through TCM has exhibited a steady growth trend in recent years. Between 2014 and 2018, the increase in the number of related studies was relatively slow; however, since 2019, the number of publications in this field has grown significantly, with 78 relevant papers published in 2023. Notably, as of 2024, 79 relevant publications have already been published this year, indicating that this research area continues to garner widespread academic attention.

An analysis based on the corresponding authors’ countries revealed that China produced the highest number of related studies (*n* = 268), far exceeding those of other countries. It was followed by the United States (*n* = 17), South Korea (*n* = 9), Japan (*n* = 8), and Italy (*n* = 5). It is noteworthy that among the top five countries in terms of publication output, the proportions of multiple-country publications (MCPs) for China and Japan were only 5.6% and 0%, respectively—significantly lower than those for the United States (41.2%) and South Korea (44.4%) (Fig. 2B; Table 1).

**Table 1:** Most relevant countries by corresponding authors

Country	Articles	SCP	MCP	Freq (%)	MCP (%)
China	268	253	15	77.46	5.6
USA	17	10	7	4.91	41.2
Korea	9	5	4	2.60	44.4
Japan	8	8	0	2.31	0
Italy	5	3	2	1.45	40
Poland	5	4	1	1.45	20
Brazil	4	1	3	1.16	75
Thailand	4	3	1	1.16	25
Iran	3	2	1	0.87	33.3
Romania	3	3	0	0.87	0
Spain	3	2	1	0.87	33.3
Australia	2	1	1	0.58	50
France	2	1	1	0.58	50
Germany	2	1	1	0.58	50
India	2	2	0	0.58	0
New Zealand	2	2	0	0.58	0
Denmark	1	0	1	0.29	100
Egypt	1	0	1	0.29	100
Mexico	1	1	0	0.29	0
Netherlands	1	1	0	0.29	0

Note: MCP: Multiple country publication; SCP: Single country publication

**Fig. 3:**
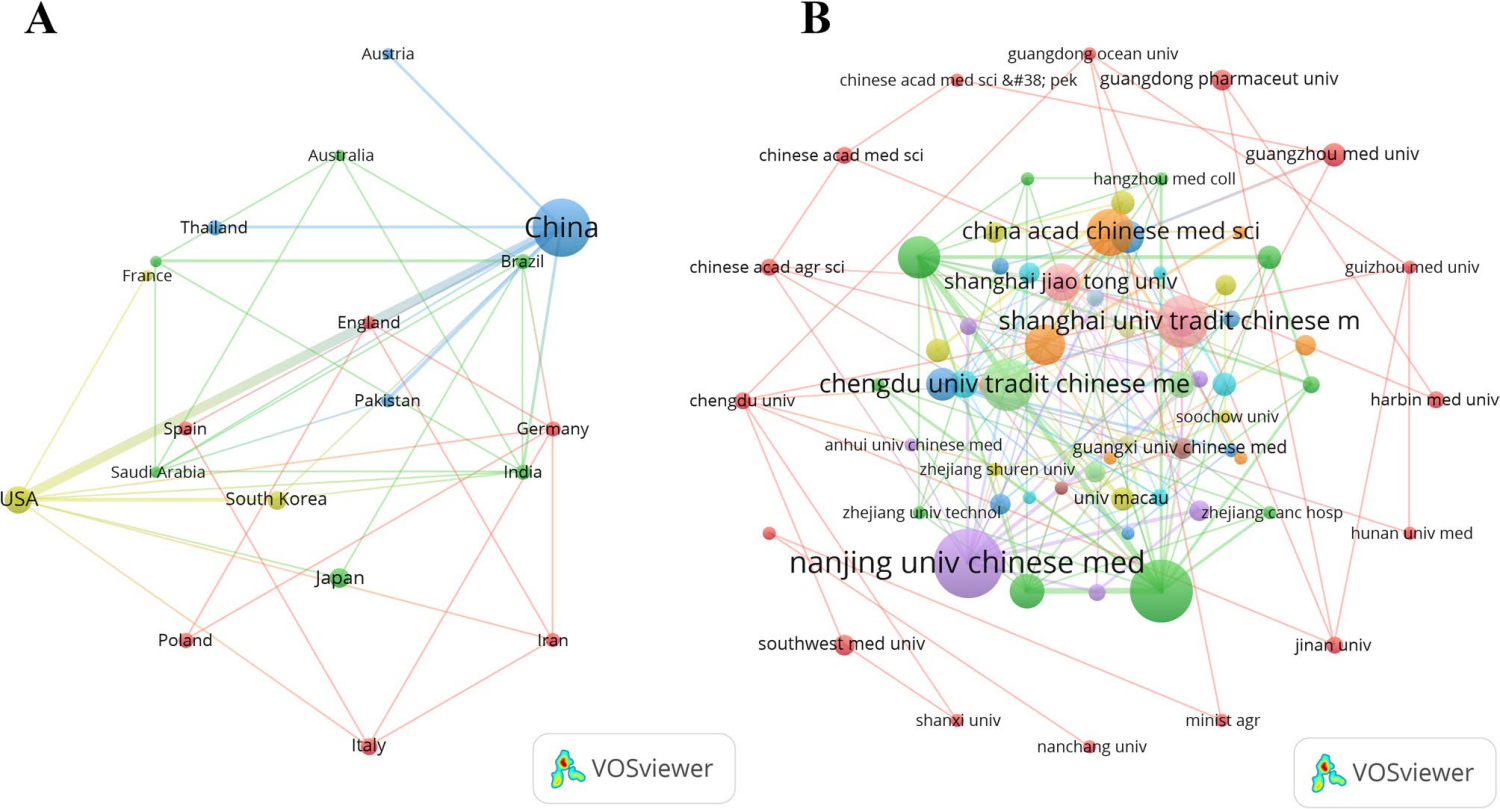
Map of countries/regions and institutions The Relationship Between MedDiet and DM from 2014 to 2024. (**A**) Map of cooperation between different countries. (**B**) Map of cooperation between different institutions

Furthermore, Fig. 3A demonstrates that in the field of cancer treatment by modulating the gut microbiota through TCM, China has established extensive academic collaboration networks with multiple countries. An analysis of collaborative institutions indicated that Nanjing University of Chinese Medicine (*n* = 60) and Chengdu University of Traditional Chinese Medicine (*n* = 44) have played central roles in research collaborations in this field (Fig. 3B; Table 2).

**Table 2:** Most relevant affiliations involved in TCM - based gut microbiota regulation for Cancer treatment

Affiliation	Articles(*n*)
NANJING UNIVERSITY OF CHINESE MEDICINE	60
CHENGDU UNIVERSITY OF TRADITIONAL CHINESE MEDICINE	44
SHANGHAI UNIVERSITY OF TRADITIONAL CHINESE MEDICINE	42
ZHEJIANG CHINESE MEDICAL UNIVERSITY	39
CHINESE ACADEMY OF SCIENCES	24
CHINA ACADEMY OF CHINESE MEDICAL SCIENCES	22
BEIJING UNIVERSITY OF CHINESE MEDICINE	17
SHANDONG UNIVERSITY OF TRADITIONAL CHINESE MEDICINE	15
GUANGZHOU UNIVERSITY OF CHINESE MEDICINE	14
MACAU UNIVERSITY OF SCIENCE AND TECHNOLOGY	14
ZHEJIANG UNIVERSITY	14
JIANGNAN UNIVERSITY	13
CHINESE ACADEMY OF MEDICAL SCIENCES - PEKING UNION MEDICAL COLLEGE	12
GUANGZHOU MEDICAL UNIVERSITY	12
SHANGHAI JIAO TONG UNIVERSITY	12
TIANJIN UNIVERSITY OF TRADITIONAL CHINESE MEDICINE	12
UNIVERSITY OF MICHIGAN	11
UNIVERSITY OF MICHIGAN SYSTEM	11
HUNAN UNIVERSITY OF CHINESE MEDICINE	10
SICHUAN UNIVERSITY	10
CAPITAL MEDICAL UNIVERSITY	9
SHAANXI UNIVERSITY OF CHINESE MEDICINE	9
UNIVERSITY OF CHINESE ACADEMY OF SCIENCES, CAS	9
XIYUAN HOSPITAL, CACMS	9
CHANGCHUN UNIVERSITY OF CHINESE MEDICINE	8

### Journal analysis and visualization

In this study, we utilized the Bibliometrix and ggplot2 packages within R software (version 3.6.3) to analyze the journals with the highest publication output and those with the highest citation frequency in this field. Additionally, VOSviewer (version 1.6.17) was employed to analyze co-cited journals. The results indicated that the 347 articles were distributed among 149 academic journals (Annex 2).

**Fig. 4:**
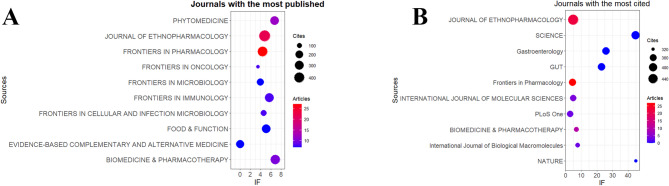
Journal with the largest number of articles published and the journal with the largest number of citations. (**A**) Journal with the largest number of articles published. (**B**) Journals with the largest number of citations

According to the publication count statistics (Table 3; Fig. 4A), *Frontiers in Pharmacology* is the journal with the highest number of publications, with a total of 27 related articles and an impact factor (IF) of 4.4. It was followed by *Journal of Ethnopharmacology* and *Phytomedicine*, which published 22 (IF = 4.8) and 12 (IF = 6.7) articles, respectively. Other relatively active journals included *Biomedicine & Pharmacotherapy* and *Frontiers in Cellular and Infection Microbiology*, which published 10 (IF = 6.9) and 8 (IF = 4.6) articles, respectively.

**Table 3:** Top 10 journals with the most published

Sources	Documents	IF (2023)	Cites
FRONTIERS IN PHARMACOLOGY	27	4.4	368
JOURNAL OF ETHNOPHARMACOLOGY	22	4.8	471
PHYTOMEDICINE	12	6.7	256
BIOMEDICINE & PHARMACOTHERAPY	10	6.9	330
FRONTIERS IN CELLULAR AND INFECTION MICROBIOLOGY	8	4.6	120
FRONTIERS IN IMMUNOLOGY	8	5.7	288
FRONTIERS IN ONCOLOGY	8	3.5	82
EVIDENCE-BASED COMPLEMENTARY AND ALTERNATIVE MEDICINE	7	0	219
FOOD & FUNCTION	7	5.1	275
FRONTIERS IN MICROBIOLOGY	7	4	177

**Fig. 5:**
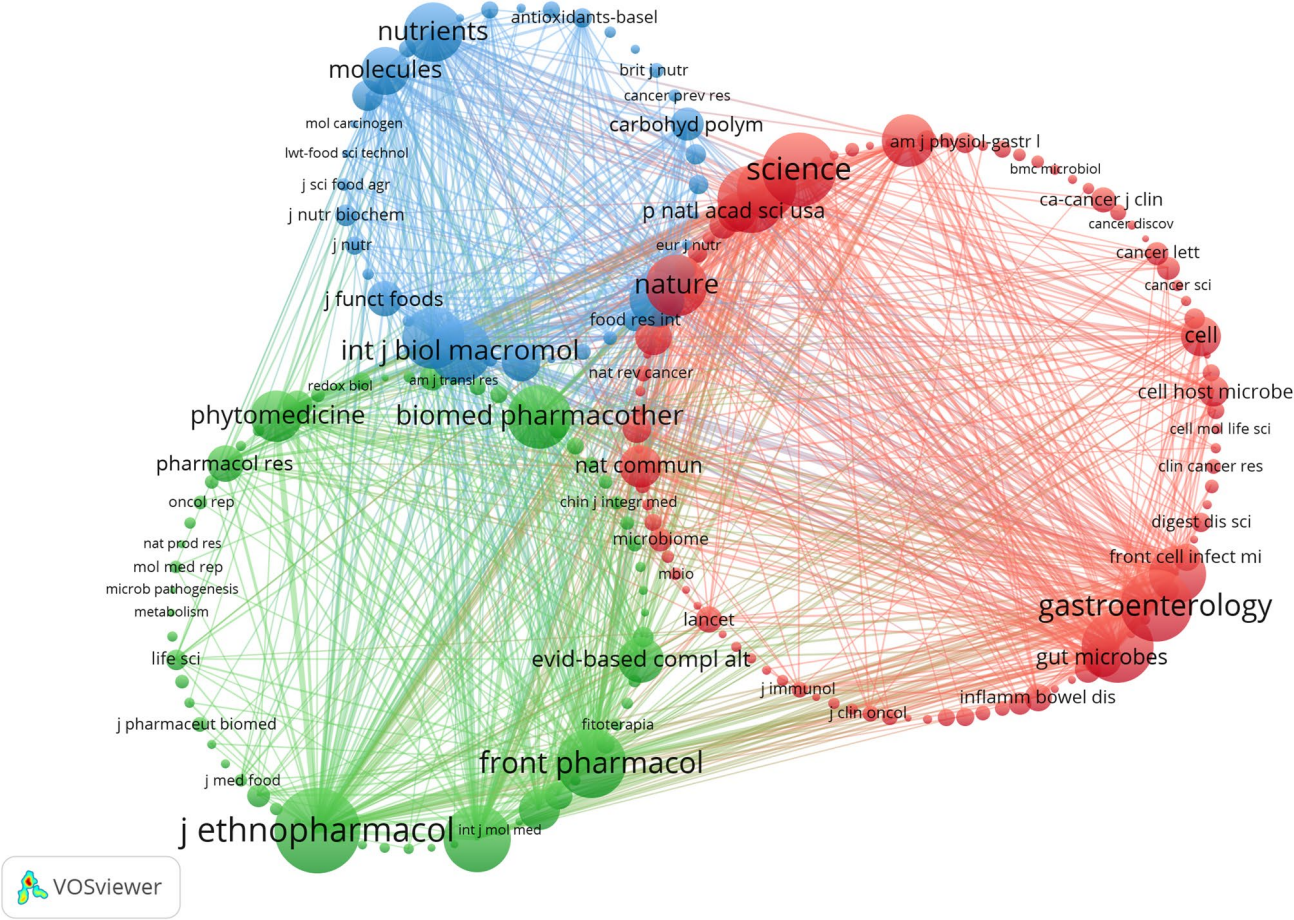
Co-cited Journals Involved in the Relationship Between TCM and Cancer Treatment via Gut Microbiota Modulation

Further analysis of citation frequency (Table 4; Fig. 4B) revealed that *Journal of Ethnopharmacology* had the highest number of citations in this field, totaling 471. It was followed by *Science* (397 citations, IF = 44.7), *Gastroenterology* (379 citations, IF = 25.7), *Gut* (378 citations, IF = 23), and *Frontiers in Pharmacology* (368 citations, IF = 4.4). Notably, both *Journal of Ethnopharmacology* and *Frontiers in Pharmacology* led in terms of publication output and citation frequency, suggesting that they may be the most representative journals in this field. In the co-citation journal map (Fig. 5), these two journals also appeared as core collaborative journals.

**Table 4:** Top 10 journals with the most cited

Sources	Cites	IF (2023)	Documents
JOURNAL OF ETHNOPHARMACOLOGY	471	4.8	22
SCIENCE	397	44.7	0
Gastroenterology	379	25.7	0
GUT	378	23	0
Frontiers in Pharmacology	368	4.4	27
INTERNATIONAL JOURNAL OF MOLECULAR SCIENCES	351	4.9	4
PLoS One	350	2.9	3
BIOMEDICINE & PHARMACOTHERAPY	330	6.9	10
International Journal of Biological Macromolecules	327	7.7	3
NATURE	320	50.5	0

These findings indicate that *Journal of Ethnopharmacology* and *Frontiers in Pharmacology* are likely the most influential journals in the current research landscape. However, the analysis also revealed that the number of publications in top-tier journals is relatively low. This phenomenon further suggests that current research has not yet achieved a sufficiently high level of academic rigor, highlighting the need for future studies to enhance the depth and quality of research in this field.

### Bursts of references

We used the bibliometrix package in R software to identify the top 20 most-cited references in this field (see Table 5). All of these references have been cited more than 85 times and originate from 18 different journals, indicating that the research in this area has not yet achieved groundbreaking progress. Notably, no single dominant journal is represented among these top 20 highly cited works. The three most-cited articles are: *Human Gut Microbiota and Gastrointestinal Cancer*, *Ganoderma lucidum Polysaccharide Modulates Gut Microbiota and Immune Cell Function to Inhibit Inflammation and Tumorigenesis in Colon*, and *Natural Product Interventions for Chemotherapy and Radiotherapy-Induced Side Effects*.

**Table 5:** Top 20 most cited references on TCM’s regulation of gut microbiota in Cancer treatment

Paper	DOI	Total Citations	TC per Year
MENG CT, 2018, GENOM PROTEOM BIOINF	10.1016/j.gpb.2017.06.002	272	34
GUO CL, 2021, CARBOHYD POLYM	10.1016/j.carbpol.2021.118231	227	45.4
ZHANG QY, 2018, FRONT PHARMACOL	10.3389/fphar.2018.01253	216	27
WENG WH, 2022, SEMIN CANCER BIOL	10.1016/j.semcancer.2020.02.011	182	45.5
LAVEFVE L, 2020, FOOD FUNCT	10.1039/c9fo01634a	171	28.5
CHEN L, 2022, CRIT REV FOOD SCI	10.1080/10408398.2021.1917508	167	41.75
REN YL, 2018, J NUTR BIOCHEM	10.1016/j.jnutbio.2018.03.005	151	18.88
LV J, 2019, CELL DEATH DIS	10.1038/s41419-019-1638-6	149	21.29
HAN K, 2021, NAT BIOMED ENG	10.1038/s41551-021-00749-2	135	27
HU QC, 2021, PHARMACOL RES	10.1016/j.phrs.2021.105444	125	25
HU Y, 2016, CARCINOGENESIS	10.1093/carcin/bgw019	125	12.5
CHEN LL, 2018, CARCINOGENESIS	10.1093/carcin/bgy009	118	14.75
ASHKTORAB H, 2019, NUTRIENTS	10.3390/nu11050943	114	16.29
SMIRNOV KS, 2016, INT J MED MICROBIOL	10.1016/j.ijmm.2016.03.006	108	10.8
FENG XC, 2020, PHARMACOL THERAPEUT	10.1016/j.pharmthera.2020.107650	99	16.5
ZHU F, 2017, TRENDS FOOD SCI TECH	10.1016/j.tifs.2016.12.003	98	10.89
LIU Y, 2022, J ETHNOPHARMACOL	10.1016/j.jep.2022.115084	97	24.25
LIU BH, 2020, FRONT PHARMACOL	10.3389/fphar.2020.01036	95	15.83
WANG CZ, 2015, INT J ONCOL	10.3892/ijo.2015.3173	95	8.64
ZHANG YP, 2018, BIOMED PHARMACOTHER	10.1016/j.biopha.2018.03.158	87	10.88

A detailed analysis of these 20 references reveals that while some articles delve into the mechanisms by which TCM treats cancer via modulation of the gut microbiota—demonstrating that TCM polysaccharides and herbal formulations can alter gut microbiota composition to exert anti-cancer effects through inhibiting inflammation, modulating immune responses, and inducing apoptosis via various signaling pathways (with some findings validated in both in vitro and in vivo experiments)—most of the literature does not elaborate in depth on how TCM specifically regulates the gut microbiota to combat cancer.

**Fig. 6:**
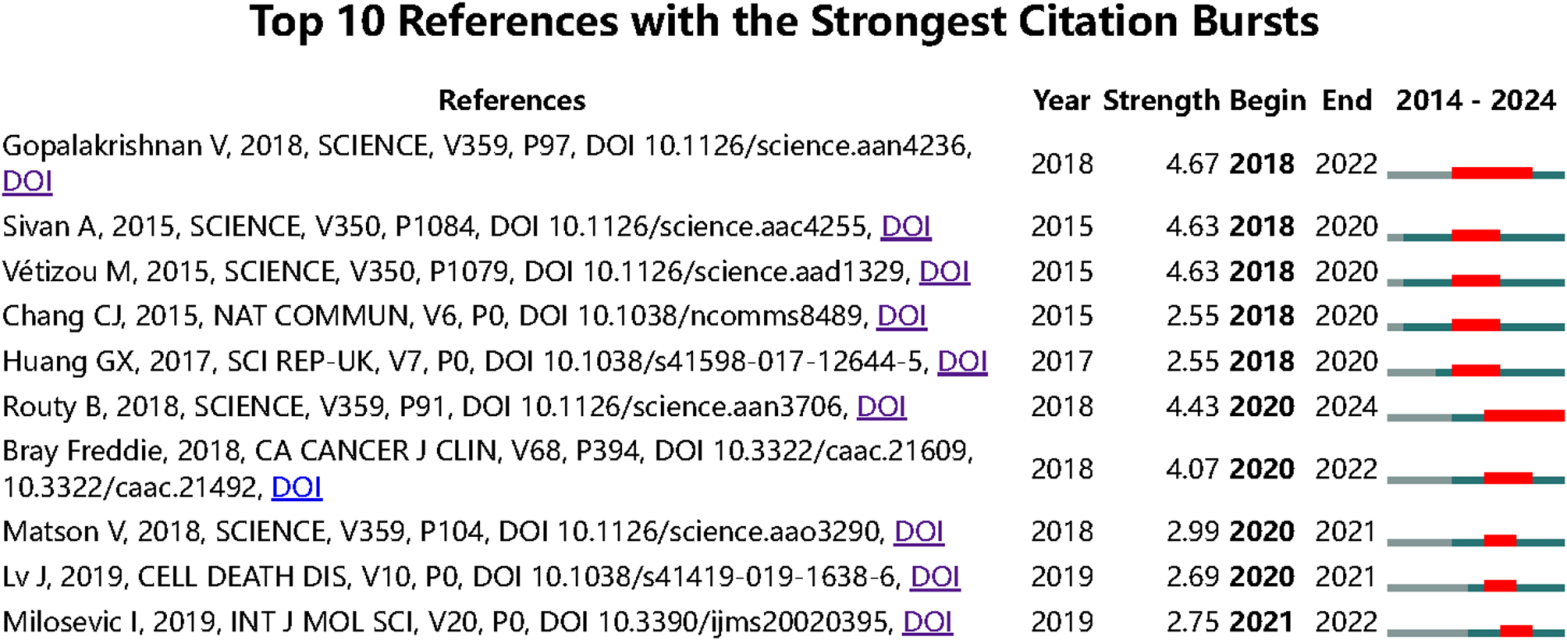
Top 10 References with the Strongest Citation Bursts on the Role of TCM in Cancer Treatment via Gut Microbiota Modulation

To identify the most influential co-cited burst references in the field of TCM-based cancer therapy, we employed CiteSpace software (screening criteria: top 10; state count: 2; minimum duration: 2). The results revealed 10 references with the strongest co-citation bursts (Fig. 6). Among these, *Gut Microbiome Modulates Response to Anti–PD-1 Immunotherapy in Melanoma Patients* (burst strength: 4.67), *Commensal Bifidobacterium Promotes Antitumor Immunity and Facilitates Anti–PD-L1 Efficacy* (burst strength: 4.63), and *Anticancer Immunotherapy by CTLA-4 Blockade Relies on the Gut Microbiota* (burst strength: 4.63) ranked in the top three.

The three most cutting-edge publications among these include *Gut Microbiome Influences Efficacy of PD-1–Based Immunotherapy Against Epithelial Tumors*, *Gut-Liver Axis*,* Gut Microbiota*,* and Its Modulation in the Management of Liver Diseases: A Review of the Literature*, and *Global Cancer Statistics 2018: GLOBOCAN Estimates of Incidence and Mortality Worldwide for 36 Cancers in 185 Countries*.

To further understand the research frontiers and hotspots in this field, we matched the DOIs of the 10 references in Fig. 6 with the titles listed in Annex 3. The results indicate that studies on the relationship between immunotherapy and the gut microbiota dominate the field—especially those investigating the interplay between immune checkpoint inhibitors (e.g., PD-1, CTLA-4) and the gut microbiota, which account for approximately 40% of the literature. Research exploring the combined use of TCM and immunotherapy accounts for about 20%.

These findings suggest that the gut microbiota plays a critical role in cancer immunotherapy, particularly in influencing the response to PD-1/PD-L1 inhibitors. Some studies have found that the diversity of the gut microbiota and the abundance of specific bacterial species are closely linked to the efficacy of immunotherapy. However, changes in the gut microbiota largely depend on patients’ lifestyles and concurrent medication regimens during clinical treatment, which may raise concerns about the reproducibility of gut microbiota intervention studies. With the growing attention on the gut microbiota, the precise modulation of gut microbiota to enhance immunotherapy efficacy has emerged as an important direction for future research.

### Keyword clusters and evolution of themes

Keyword clustering is an important tool for understanding the hot topics and developmental directions within a research field. In this study, we used VOSviewer to extract a total of 1,095 keywords, excluding overly common terms such as “traditional Chinese medicine”, “cancer”, and “gut microbiota” (see Table 6). The top 20 keywords, each appearing more than 15 times, include: inflammation (*n* = 44), colorectal cancer (*n* = 43), cells (*n* = 41), expression (*n* = 29), mice (*n* = 29), inflammatory bowel disease (*n* = 27), NF-kappa B (*n* = 23), and chain fatty acids (*n* = 22).

**Table 6:** The top 20 keywords on TCM’s regulation of gut microbiota in Cancer treatment

Rank	Keywords	Count
1	inflammation	44
2	colorectal-cancer	43
3	cells	41
4	expression	29
5	mice	29
6	inflammatory-bowel-disease	27
7	nf-kappa-b	23
8	chain fatty-acids	22
9	disease	22
10	in-vitro	22
11	activation	20
12	oxidative stress	20
13	colitis	19
14	growth	18
15	ulcerative-colitis	18
16	apoptosis	17
17	bacteria	17
18	dysbiosis	17
19	inhibition	17
20	mechanisms	16

**Fig. 7:**
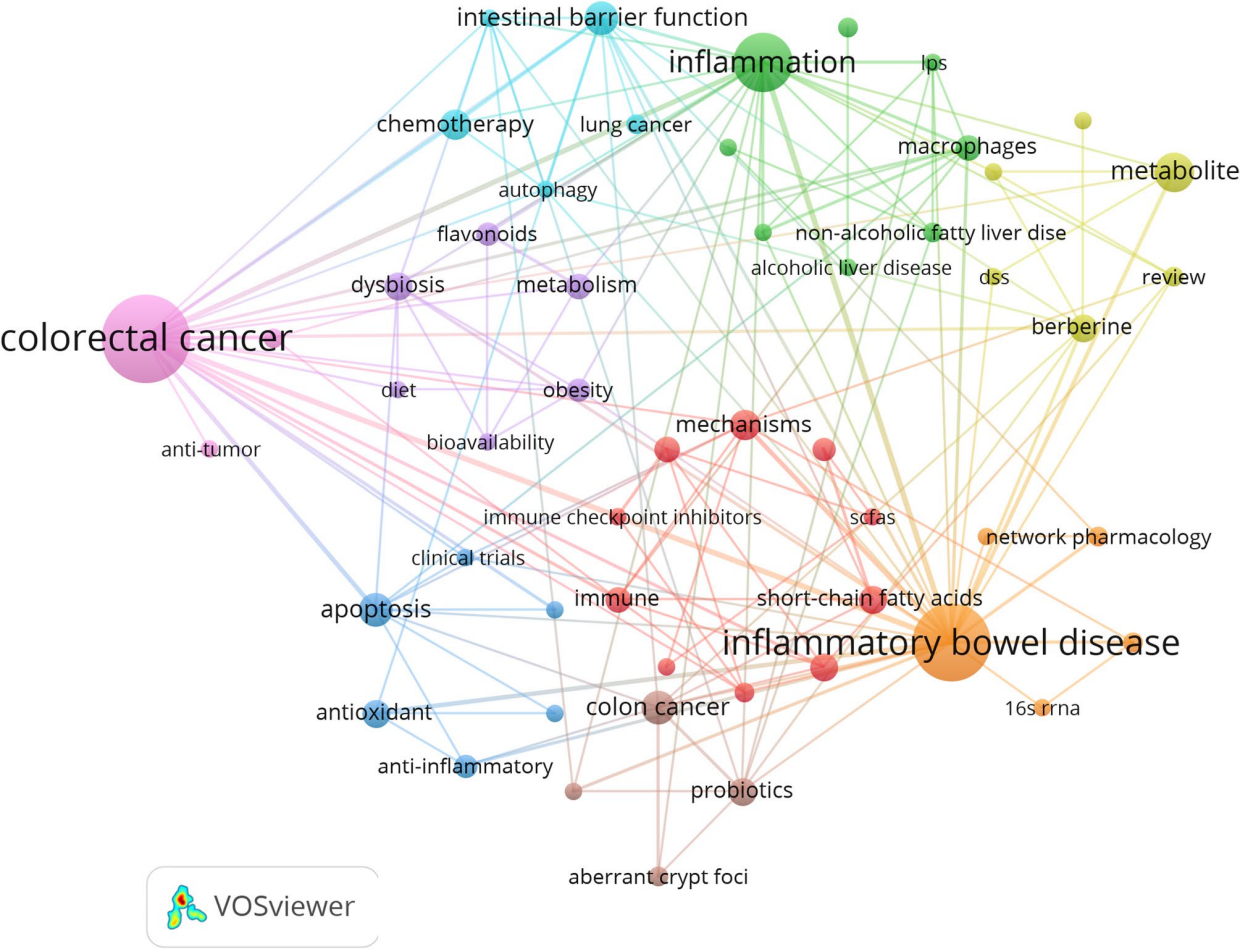
Keyword Co-occurrence Map of Publications on the Role of TCM in Cancer Treatment via Gut Microbiota Modulation

Subsequently, using a threshold of “minimum keyword occurrence ≥ 3,” a total of 55 keywords were selected for the construction of a keyword clustering map **(**Fig. 7**)**. This map reveals nine distinct clusters, each represented by a different color and corresponding to various research domains.

The examination of gut microbiota and its interactions with diverse biological systems unveils complex relationships spanning multiple research fields. The Red Cluster includes 10 keywords, focusing on the connection between gut microbiota and immunity, with terms like fecal microbiota transplantation, immune checkpoint inhibitors, immunotherapy, and related mechanisms. The Green Cluster includes 8 keywords, exploring the interactions between liver diseases and gut inflammatory responses, including alcoholic liver disease, colitis-associated cancer, gut-liver axis, and lipopolysaccharide. The Dark Blue Cluster includes 6 keywords, investigating anti-inflammatory and antioxidant properties, highlighting clinical trials and key components like apoptosis and Escherichia coli. The Yellow Cluster includes 6 keywords, delving into metabolism, particularly lipid metabolism, exploring berberine, DSS (dextran sodium sulfate), liver injury, and metabolites. The Purple Cluster includes 6 keywords, examining nutrition and its impact on metabolism, with an emphasis on bioavailability, diet, dysbiosis, flavonoids, and metabolism. The Light Blue Cluster includes 5 keywords, dedicated to cancer treatment and its mechanisms, investigating topics like autophagy, chemotherapy, curcumin, intestinal barrier function, and lung cancer. The Orange Cluster includes 5 keywords, focusing on gut health and function, covering inflammatory bowel disease, irritable bowel syndrome, network pharmacology, and polyphenols. The Brown Cluster includes 3 keywords, highlighting cancer-related natural products, including aberrant crypt foci, aloe vera, and colon cancer. Finally, the Pink Cluster includes 3 keywords, emphasizing antitumor activities, specifically in colorectal cancer and Fusobacterium nucleatum. These clusters illustrate the diverse yet interconnected role of microbiota, metabolism, and disease mechanisms across various physiological contexts. (Annex 4).

**Fig. 8:**
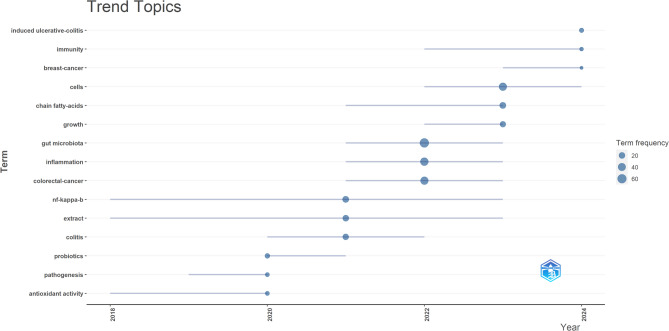
Trend Topics in TCM’s Role in Cancer Therapy through Gut Microbiota Modulation

In addition, we employed the Bibliometrix package in R software to generate a thematic evolution map. This map effectively illustrates the temporal evolution of specific research topics in the field, helping to analyze the dynamic trends. By examining Fig. 8, we were able to clearly delineate the research focuses and evolution trajectories in the field of cancer treatment through the modulation of gut microbiota using TCM.

Our findings reveal that the gut microbiota can influence cancer cell growth, apoptosis, and immune evasion by affecting the host’s immune system, inflammatory status, and metabolic processes, particularly in cancers such as colorectal and breast cancer. Gut microbial metabolites—such as short-chain fatty acids—can inhibit inflammation by modulating signaling pathways like NF-κB, thereby suppressing tumor progression. TCM and its active components, such as berberine, curcumin, and polyphenolic compounds, significantly enhance antitumor effects by improving the composition of the gut microbiota and promoting the generation of beneficial metabolites. Moreover, microbial intervention strategies, including the use of probiotics and fecal microbiota transplantation, have been applied to enhance the efficacy of cancer immunotherapy. The mechanisms by which TCM modulates the gut microbiota offer new perspectives for precision cancer treatment in the future.

Overall, the keyword clustering and trend analysis indicate that research hotspots in this field mainly focus on the regulation of gut microbiota by TCM, particularly in relation to improving inflammation and metabolic dysregulation. These studies hold potential for the adjuvant treatment or prevention of cancer. As research continues to advance, the role of TCM in modulating the gut microbiota for cancer treatment is expected to become increasingly prominent, forming an integral component of future cancer therapies.

## Discussion

### General information

In this study, we selected relevant literature from databases, comprising 347 publications from 2014 to 2024. The publication trends indicate that the number of studies on the regulation of gut microbiota by TCM for cancer treatment has been steadily increasing. From 2014 to 2018, the number of related publications showed a slow upward trend. However, from 2019 to 2023, the number of publications grew rapidly, with 78 papers published in 2023. As of 2024, 79 related studies have already been published. This trend suggests that research interest in this field is rising, potentially due to the following three reasons: (a) Increasing Interest in the Field: In recent years, numerous studies have revealed the critical role of gut microbiota in regulating tumor immunity, metabolism, and drug resistance. For example, a study by Yang Li et al. demonstrated the potential mechanisms by which TCM modulates gut microbiota to improve immune function and intestinal barrier integrity in colorectal cancer patients [35]. This provides a theoretical basis for utilizing TCM to regulate gut microbiota in cancer treatment.(b) Advancements in Research Technologies: With the rapid development of research techniques, scientists can now more precisely analyze the complex interactions between active compounds in TCM and gut microbiota. A comparative proteomics study has identified potential targets of TCM active components in modulating intracellular signaling pathways in cancer cells, thereby advancing research in this field [36, 37].(c) Integration of TCM with Modern Medicine: As research progresses, the principles of TCM increasingly align with modern oncology theories. For instance, TCM’s ability to reshape gut microbiota and the tumor microenvironment complements modern immunotherapy approaches, leading to more effective cancer treatments [35, 38].

China has emerged as the leading country in this research domain, publishing the highest number of academic papers on TCM-mediated modulation of gut microbiota for cancer therapy. This trend reflects the deep historical significance of TCM in China and the substantial attention it has received from Chinese researchers.

The 347 publications are distributed across 149 different journals, with *Frontiers in Pharmacology*, *Journal of Ethnopharmacology*, and *Phytomedicine* being the most prominent. Notably, *Journal of Ethnopharmacology* and *Frontiers in Pharmacology* have published a significant number of highly cited papers. Their outstanding performance highlights their role as key publication platforms in this field, serving as primary channels for disseminating research findings on TCM-based gut microbiota regulation in cancer treatment.

### Hotspots and development trends

Through literature clustering, keyword frequency analysis, keyword clustering, and thematic evolution analysis, we identified the potential research hotspots in the area of TCM regulating gut microbiota for cancer treatment. The research results show that the frontiers and hotspots in this field mainly focus on three aspects. First, The types of cancer that TCM can treat through gut microbiota. Second, the extracts of TCM that regulate gut microbiota to treat cancer. Third, how TCM targets specific bacterial populations in the gut microbiota for cancer treatment.

#### The types of cancer that TCM can treat through gut microbiota

Through analysis, we found that the cancers most studied in relation to TCM regulating gut microbiota for treatment mainly focus on colorectal cancer, liver cancer, gastric cancer, and other cancers [39]. Previous studies have indicated that gut microbiota dysbiosis is highly likely to lead to the occurrence of colorectal cancer [19, 40, 41]. For example, Escherichia coli carrying the pks pathogenic island (pks + E. coli) induces cellular senescence by regulating SENP1 expression and adjusts the levels of miR-20a-5p and related growth factors, thereby promoting cell proliferation and tumorigenesis [42]. In addition, Fusobacterium nucleatum promotes the progression of colorectal cancer (CRC) through various mechanisms. First, Fusobacterium nucleatum plays a key promoting role in CRC progression, involving multiple aspects. In terms of metabolism, Fusobacterium nucleatum targets the α-enolase 1 pathway to enhance glucose metabolism in CRC cells, providing sufficient energy and material support for tumor cell proliferation and survival, thus promoting carcinogenesis [43]. In immune regulation, Fusobacterium nucleatum regulates Th17 immune responses in a free fatty acid receptor 2-dependent manner, which influences the formation of a pro-cancerous gut microenvironment [44]. In signal pathway activation, Fusobacterium nucleatum activates the NF-κB signaling pathway in CRC cells, leading to reduced miR-1322 expression, CCL20 activation, macrophage infiltration, and M2 macrophage polarization, which promote tumor immune evasion and the inflammatory response in the tumor microenvironment [45]. Additionally, the metabolic products of Fusobacterium nucleatum, such as formate, induce aryl hydrocarbon receptor signaling, driving CRC tumor invasion and increasing cancer stemness [46]; another metabolite activates the Alpha-kinase 1 /TIFA adaptor protein/Tumor Necrosis Factor Receptor Associated Factor 6 pathway to activate the NF-κB pathway, enhancing inflammatory cytokine and anti-apoptotic gene expression, promoting tumor cell survival and proliferation [47].

Lactic acid bacteria (L.) and their metabolites play an important role in restricting colon tumor growth [48]. Lactic acid bacteria achieve this through the following mechanisms: enhancing CD8 T cell recruitment by interacting with immune cells via specific signaling mechanisms, activating related signaling pathways, and guiding CD8 T cells to migrate and accumulate in the tumor microenvironment, thereby strengthening the body’s anti-tumor immune response and inhibiting the proliferation and growth of colon tumor cells [49]. In terms of redox balance, lactic acid bacteria regulate the intracellular redox state through their own metabolic processes, maintaining cellular homeostasis and reducing oxidative stress-induced damage to biological macromolecules like DNA, thus inhibiting tumor cell initiation and progression [50]. By increasing dendritic cell production of IL-12a, lactic acid bacteria interact with dendritic cells to activate specific transcription factors and signaling pathways, promoting IL-12a gene transcription and protein synthesis. IL-12a then further activates natural killer cells and T cells, enhancing the body’s anti-tumor immune capacity [48]. Current research suggests that TCM extracts can intervene in the occurrence of colorectal cancer by regulating lactic acid bacteria and their metabolites [51, 52].

In the case of gastric cancer, the gut-liver axis regulates the interaction between gut microbiota and liver cancer through the lymphatic system, portal vein, and bile circulation system [53]. Approximately 75% of liver blood supply comes from the portal vein, carrying gut bacteria and their metabolites, i.e., microbial-associated molecular patterns (MAMPs) [54]. Metabolites produced by anaerobic bacteria in the colon can damage the intestinal barrier and promote the entry of MAMPs into the liver. These substances are absorbed by the liver and excreted back into the intestine via bile, forming the gut-liver circulation and continuously affecting the interaction between the gut and liver [55].

Deoxycholic acid (DCA), a product of bile acid transformation by gut microbiota, plays a key role in the development of liver cancer related to gut microbiota. DCA promotes hepatocellular carcinoma (HCC) through multiple mechanisms, such as DCA synthesis producing reactive nitrogen and oxygen species, which induce DNA damage and lead to the occurrence of HCC. Furthermore, DCA’s gut-liver circulation induces a senescence-associated secretory phenotype in hepatic stellate cells, leading to the secretion of inflammatory and pro-tumor factors from the liver, thereby promoting HCC progression [56, 57]. Existing studies have found that TCM mixtures can improve the development of liver cancer by regulating gut microbiota [58]. Additionally, other studies suggest that TCM or TCM extracts can significantly prevent the development of various cancers.

However, there are some limitations in such studies: (a) Lack of depth in mechanistic research: Although studies have explored the mechanisms by which TCM regulates gut microbiota, there is still a lack of in-depth research into the specific processes by which TCM inhibits cancer through gut microbiota modulation. TCM is complex, and there may be interactions between various active ingredients, which increases the complexity of research and the difficulty of interpreting results. (b) Lack of clinical studies: Most research remains at the laboratory stage, with a lack of large-scale clinical trials to verify the effectiveness and safety of TCM in regulating gut microbiota for cancer treatment. Existing clinical studies are limited, mostly small-scale, short-term studies, and lack long-term follow-up and validation with large sample sizes. (c) Diversity in research methods: Different studies use different methods and models, resulting in poor comparability and consistency of results, which affects the reliability of research conclusions. There is currently a lack of unified research standards and evaluation systems, making it difficult to interpret and compare research findings.

#### Traditional Chinese medicine extracts in the regulation of gut microbiota for cancer treatment

A comprehensive review of the literature reveals that research efforts have predominantly concentrated on traditional Chinese medicinal herbs and their bioactive extracts, specifically licorice polysaccharides, ginseng polysaccharides, polyphenols, and flavonoids, for modulating the gut microbiota within cancer therapeutic frameworks. Among these, licorice polysaccharide (GCP), a high-molecular-weight polysaccharide extracted from the traditional Chinese medicine licorice, has been demonstrated to inhibit tumor growth both in vitro and in vivo. GCP modulates the expression of tumor-related genes such as Bcl-2 and Bax, thereby inducing apoptosis in tumor cells and inhibiting tumor formation and growth. In addition, GCP exerts its anti-tumor effects by enhancing immune function, promoting lymphocyte proliferation, and increasing macrophage activity. Moreover, GCP may further contribute to its anti-cancer effects by modulating the composition of the gut microbiota [59, 60].

Recent studies have also demonstrated that ginseng polysaccharides can modulate the composition of the gut microbiota. For example, by increasing the abundance of lactic acid bacteria and bifidobacteria, these polysaccharides enhance immune function and exert anticancer effects [61]. Additionally, flavonoids represent a large class of natural compounds with anti-cancer activity. Among them, naringin, as a representative component, exhibits significant anti-cancer effects while displaying low toxicity toward normal cells. Research has indicated that the modulation of specific gut microbes can be employed in the treatment of epithelial ovarian cancer (EOC) [62, 63].

Polyphenolic compounds, which are present in various traditional Chinese herbs, have also shown a significant ability to modulate the gut microbiota. Studies suggest that these compounds can alter the gut microbial community by promoting the growth of beneficial bacteria such as Lactobacillus and Bifidobacterium while inhibiting harmful bacteria. For example, baicalin, extracted from Scutellaria baicalensis, has been demonstrated to improve intestinal barrier function and modulate inflammatory responses, thereby influencing cancer progression [64, 65]. These polyphenols can also target cancer cells through multiple mechanisms, including the induction of apoptosis, inhibition of angiogenesis, and suppression of metastasis.

Collectively, these studies indicate that TCM and its extracts can significantly enhance the efficacy of cancer treatment by modulating the gut microbiota.

#### TCM targets specific bacterial populations in the gut microbiota to treat cancer

Recent studies have identified one of the key research focuses in this field as exploring how TCM can treat cancer by influencing specific gut microbiota populations. In the context of cancer treatment, TCM’s regulation of the gut microbiota primarily involves two categories of microbial populations:

One category consists of beneficial probiotics that contribute to anti-inflammatory and immune-modulating effects, such as Bifidobacterium, Lactobacillus, and Clostridium butyricum. These microbiota promote the production of short-chain fatty acids (SCFAs) [66], which enhance gut barrier function and regulate the immune microenvironment [67].

The other category involves pro-inflammatory bacteria associated with inflammation and tumorigenesis. Some Chinese herbal medicines may play a role in cancer treatment by inhibiting the abundance of Fusobacterium [47] and certain Bacteroides strains [68], thereby reducing the production of pro-inflammatory metabolites.

Furthermore, recent research suggests that Ackermannia plays a positive role in enhancing the efficacy of immune checkpoint inhibitors and restoring gut barrier function. Traditional Chinese herbs such as patchouli and Xini decoction have been shown to increase the abundance of Ackermannia, thus improving its auxiliary role in cancer treatment [69, 70].

Although growing evidence supports the idea that TCM can improve cancer treatment outcomes by regulating the gut microbiota, further research is needed. The current experimental studies have the following limitations: (a) The existing evidence primarily comes from in vitro experiments or animal studies, with a lack of large-scale, randomized controlled clinical trials to validate the therapeutic effects of these microbial regulatory actions in cancer patients.(b) The composition of the gut microbiota is complex and highly influenced by individual differences, diet, lifestyle, and other factors. This makes it difficult to standardize and replicate the effects of TCM in regulating specific microbiota, potentially leading to significant variability in treatment outcomes across patients.(c) While studies have shown that Ackermannia and other bacterial species are associated with immune therapy efficacy, the interactions between these species and other gut microbiota, as well as the potential overall ecological changes induced by TCM in modulating these populations, remain uncertain.(d) The synergistic effects and potential antagonistic interactions between TCM and modern cancer treatments have not been fully explored, and these factors constitute significant limitations in current research.

### Limitations and future directions

#### Limitations

It must be acknowledged that this study has certain limitations. First, as a bibliometric analysis, the data collection and processing primarily relied on the software tools employed. While this approach facilitates systematic analysis of large datasets, it cannot completely replace traditional systematic literature searches, and some bias may be present. Second, this study only included English-language literature from the WoSCC, which may have excluded significant research published in other languages or indexed in different databases. However, given the broad coverage of WoSCC in this field, it is anticipated that such omissions have a limited impact on the overall trends. Third, due to the time lag associated with citation impact, some recently published high-quality studies may not have been fully cited yet, which could affect their representation in the current analysis. As such, future research should continue to track and update the data.

Despite these limitations, this study provides valuable insights for the academic community and contributes to a more comprehensive understanding of the development trends, research hotspots, and emerging directions in the field of TCM’s role in regulating gut microbiota for cancer treatment [71]. 

#### Future directions

Although existing studies have demonstrated significant potential for TCM in regulating gut microbiota to assist in cancer treatment, the understanding of its specific biological mechanisms, patient variability, and long-term efficacy remains incomplete. Currently, most research is still at the preliminary stage of basic experiments and animal models, with clinical validation being in the exploratory phase. Future studies must systematically analyze the interactions between the active components of TCM and gut microbiota, combined with multi-center clinical trials to assess its safety and efficacy across different patient populations. Efforts should also focus on developing more targeted and personalized treatment strategies to advance the field toward precision-based applications.

## Conclusion

Our study highlights the research hotspots related to TCM in treating cancer through the regulation of gut microbiota, and elucidates key knowledge domains and emerging frontiers within this field. Specifically, our analysis identifies three major current research directions: (a) exploring the application of TCM in the treatment of various cancer types through the regulation of gut microbiota; (b) investigating how TCM extracts affect the structure of gut microbiota to exert anti-cancer effects; and (c) focusing on the potential of precision interventions targeting specific gut microbiota populations with TCM. Additionally, we discuss the existing gaps in these hotspot areas of research, arguing that addressing these issues will lay a solid foundation for future in-depth exploration and clinical application.

## Electronic supplementary material

Below is the link to the electronic supplementary material.

Supplementary Material 1

Supplementary Material 2

Supplementary Material 3

Supplementary Material 4

## Data Availability

Data will be made available on request. Data from web of science database. Available through the following URL: https://www.webofscience.com.
