# Supplementary material for: The bibliometric analysis of research on traditional Chinese medicine regulating gut microbiota for cancer treatment from 2014 to 2024
Source: Hereditas. 2025 Jun 3;162:94. doi: 10.1186/s41065-025-00456-x (PMC12131599; doi:10.1186/s41065-025-00456-x)
Supplement: Supplementary file 2 — Supplementary Material 2 [file 41065_2025_456_MOESM2_ESM.docx]

TS=("Chinese medicine" OR "traditional Chinese medicine" OR TCM OR "herbal medicine" OR "botanical medicine" OR "material medicine" OR decoction OR tang OR powder OR san OR pill OR extract OR "Chinese proprietary medicine" OR "Chinese patent medicine" OR granules OR plaster OR plasters OR "oral liquid" OR "oral liquids" OR gels OR capsule OR syrup) AND TS=("gut" OR "bowel" OR "colon" OR "intestine" OR "gastrointestinal" OR "gastro-intestinal*" OR "intestinal tract" OR "digestive tract") AND TS=("microbiome*" OR "microbiota*" OR "microflora*" OR "flora" OR "bacteria*" OR "commensal*" OR "probiotic*" OR "prebiotic*" OR "microecology" OR "dysbiosis" OR "pathobiont*" OR "symbiotic*" OR "mutualistic*" OR "pathogenic*") AND TS=("cancer*" OR "tumor*" OR "neoplasm*" OR "neoplasia*" OR "malignant neoplasm*" OR "malignancy" OR "malignancies" OR "carcinoma*" OR "sarcoma*" OR "lymphoma*" OR "leukemia*" OR "malignant tumor*" OR "metastatic cancer*") AND LA=("English") AND DOP=2014-01-01/2024-12-31 AND Preprint Citation Index (Exclude Database).
