# Supplementary material for: The bibliometric analysis of research on traditional Chinese medicine regulating gut microbiota for cancer treatment from 2014 to 2024
Source: Hereditas. 2025 Jun 3;162:94. doi: 10.1186/s41065-025-00456-x (PMC12131599; doi:10.1186/s41065-025-00456-x)
Supplement: Supplementary file 4 — Supplementary Material 4 [file 41065_2025_456_MOESM4_ESM.docx]

**Cluster1 (10)**

fecal microbiota transplantation

gastric cancer

immune

immune checkpoint inhibitors

immunotherapy

mechanisms

polysaccharides

scfas

short-chain fatty acids

tumor microenvironment

**cluster2 (8)**

alcoholic liver disease

colitis-associated cancer

gut-liver axis

inflammation

lipopolysaccharide

lps

macrophages

non-alcoholic fatty liver disease

**cluster3 (6)**

anti-inflammatory

antioxidant

apoptosis

clinical trials

escherichia coli

medicine

**cluster4 (6)**

berberine

dss

lipid metabolism

liver injury

metabolite

review

**cluster5 (6)**

bioavailability

diet

dysbiosis

flavonoids

metabolism

obesity

**cluster6 (5)**

autophagy

chemotherapy

curcumin

intestinal barrier function

lung cancer

**cluster7 (5)**

16s rrna

inflammatory bowel disease

irritable bowel syndrome

network pharmacology

polyphenols

**cluster8 (4)**

aberrant crypt foci

aloe vera

colon cancer

probiotics

**cluster9 (5)**

anti-tumor

colorectal cancer

fusobacterium nucleatum
